# Enhancing the ethanol production by exploiting a novel metagenomic-derived bifunctional xylanase/β-glucosidase enzyme with improved β-glucosidase activity by a nanocellulose carrier

**DOI:** 10.3389/fmicb.2022.1056364

**Published:** 2023-01-04

**Authors:** Shohreh Ariaeenejad, Elaheh Motamedi, Kaveh Kavousi, Rezvaneh Ghasemitabesh, Razieh Goudarzi, Ghasem Hosseini Salekdeh, Behrouz Zolfaghari, Swapnoneel Roy

**Affiliations:** ^1^Department of Systems and Synthetic Biology, Agricultural Biotechnology Research Institute of Iran (ABRII), Agricultural Research Education and Extension Organization (AREEO), Karaj, Iran; ^2^Department of Nanotechnology, Agricultural Biotechnology Research Institute of Iran (ABRII), Agricultural Research Education and Extension Organization (AREEO), Karaj, Iran; ^3^Laboratory of Complex Biological Systems and Bioinformatics (CBB), Department of Bioinformatics, Institute of Biochemistry and Biophysics (IBB), University of Tehran, Tehran, Iran; ^4^Department of Molecular Sciences, Macquarie University, Sydney, NSW, Australia; ^5^Department of Integrated Art and Sciences, Faculty of Education, Waseda University, Tokyo, Japan; ^6^School of Computing, University of North Florida, Jacksonville, FL, United States

**Keywords:** bifunctional enzyme, metagenome, Nanocellulose carrier, bioethanol, in-silico screening

## Abstract

Some enzymes can catalyze more than one chemical conversion for which they are physiologically specialized. This secondary function, which is called underground, promiscuous, metabolism, or cross activity, is recognized as a valuable feature and has received much attention for developing new catalytic functions in industrial applications. In this study, a novel bifunctional xylanase/β-glucosidase metagenomic-derived enzyme, PersiBGLXyn1, with underground β-glucosidase activity was mined by in-silico screening. Then, the corresponding gene was cloned, expressed and purified. The PersiBGLXyn1 improved the degradation efficiency of organic solvent pretreated coffee residue waste (CRW), and subsequently the production of bioethanol during a separate enzymatic hydrolysis and fermentation (SHF) process. After characterization, the enzyme was immobilized on a nanocellulose (NC) carrier generated from sugar beet pulp (SBP), which remarkably improved the underground activity of the enzyme up to four-fold at 80°C and up to two-fold at pH 4.0 compared to the free one. The immobilized PersiBGLXyn1 demonstrated 12 to 13-fold rise in half-life at 70 and 80°C for its underground activity. The amount of reducing sugar produced from enzymatic saccharification of the CRW was also enhanced from 12.97 g/l to 19.69 g/l by immobilization of the enzyme. Bioethanol production was 29.31 g/l for free enzyme after 72 h fermentation, while the immobilized PersiBGLXyn1 showed 51.47 g/l production titre. Overall, this study presented a cost-effective in-silico metagenomic approach to identify novel bifunctional xylanase/β-glucosidase enzyme with underground β-glucosidase activity. It also demonstrated the improved efficacy of the underground activities of the bifunctional enzyme as a promising alternative for fermentable sugars production and subsequent value-added products.

## Introduction

Lignocellulosic biomass can be a renewable source with great potential in bioethanol production because they are primarily agricultural waste. The most critical limiting factor for this conversion is the recalcitrant nature of lignocellulosic substrates (Nguyen et al., 2017; Kirupa Sankar et al., 2018). The major component of this lignocellulosic resource is cellulose and hemicellulose, and lignin (Duque et al., 2020). Cellulose and hemicellulose are the main components converted into sugar *via* enzymatic or chemical hydrolysis (Menezes et al., 2014). Coffee waste contains different toxic compounds, such as polyphenols, caffeine, and tannins, which makes this waste unsuitable for beneficial utilization (Leifa et al., 2000). The disposal of coffee waste into the environment negatively affects soil microorganisms and is a big concern for the processing units (Mirón-Mérida et al., 2021). Consequently, using these wastes to produce value-added materials can be economically and environmentally effective (Kim et al., 2017). In this regard, as a promising alternative biomass resource, coffee waste can potentially be subjected to biofuel and energy production (Gurram et al., 2016). Coffee is one of the most popular beverages in the world. In the process of extracting it, a significant amount of coffee residue waste (CRW) is produced, which is a non-edible agricultural by-product with a low price had been generated (Choi et al., 2012). The CRW can be a good source for bioethanol production because it comprises large amounts of polysaccharides that are fragmented to produce fermentable sugars (Triana et al., 2011; Shankar et al., 2019).

Hydrolytic enzymes are used for the biodegradation of lignocellulose during the saccharification process. Afterward, the hydrolysates obtained from the saccharification are used for ethanol production *via* fermentation. Nevertheless, in most cases, the saccharification efficiency is very poor, therefore, pretreatment methods should be applied (Sharma, 2019). During pretreatment, the recalcitrant lignocellulosic matrix is disrupted, lignin and hemicellulose fractions are degraded, and the yields of fermentable sugars are remarkably increased (Kostas et al., 2020). Some of the most used pretreatment methods are alkaline hydrolysis, liquid hot water and steam explosion (Triana et al., 2011). Among different kinds of pretreatment techniques, organic solvent pretreatment has been proven to be a promising one for the degradation of lignocellulosic biomass and biofuel applications (Zhang et al., 2016). In this method, hemicellulose is hydrolyzed almost completely with a tremendous enhancement of cellulose saccharification rate (Zhang et al., 2016).

Studies have shown that to use CRW in ethanol production, investigating the effect of different pretreatments, identifying the appropriate enzyme with proper performance, and optimizing the enzyme dosage are essential (Choi et al., 2012).

Extrusion is a successful pretreatment method in which lignocellulosic biomass is subjected to physical, chemical, compressive, and thermal pretreatments, which work synergistically to promote the degradation of the lignocellulosic matrix and the access of hydrolytic enzymes to carbohydrates (Duque et al., 2020). After the pretreatment of rice straw, hydrolytic enzymes are used to complete the saccharification, fermentation, and further ethanol production (Sharma, 2019). Most enzymes lose their activities under extreme environments, including high salinity, extreme pH, and low or high temperature. Therefore, the identification of halotolerant enzymes suited to harsh industrial conditions is highly demanded (Leadbeater, 2018).

The endoglucanases, β-glucosidases, and exoglucanases are the three most significant groups of cellulases (Menezes et al., 2014). Using β -glucosidase and xylanase simultaneously have some advantages, such as high tolerance for fermentation inhibitors that are produced by pretreated agro-waste (Takano and Hoshino, 2018). Because efficient enzymes such as β-glucosidase and xylanase act in unison, and where endo-β-1, 4-glucanases attack the internal sites of cellulose polymers and produce numerous chain ends, the chances of using these enzymes to produce biofuel are high (Patel et al., 2019). Enzymatic hydrolysis of alkali pretreated agricultural waste by cellulases and xylanases ultimately leads to high sugar production (Meneses et al., 2017).

Given that the rumen microbiota is a good source for various genes encoding lignocellulolytic enzymes, many studies have been conducted to discover new biocatalysts that hydrolyze lignocellulose (Montella et al., 2017). Metagenomic technologies facilitate the discovery of novel enzymes from rumen microbial to be used in various biotechnology industries (Ariaeenejad et al., 2021b; Patel et al., 2019; Sadeghian Motahar et al., 2021; Maleki et al., 2022). From metagenome data, previous studies identified efficient high-potential glycoside hydrolases enzymes in lignocellulose-based industries (Fatemeh et al., 2020; Maleki et al., 2020; Ariaeenejad et al., 2020b; Motamedi et al., 2021a; Ariaeenejad et al., 2021a). Bioinformatics tools and computational methods are considered accessible and fast technological techniques for achieving new enzyme sequences (Ariaeenejad et al., 2018; Foroozandeh Shahraki et al., 2020, 2021).

Enzymes are usually divided into different enzyme groups depending on what substrates they bind to and are the catalysts of the chemical reaction. However, some enzymes can bind with more than one substrate, catalyze reactions and transform more than one for which they are physiologically specialized (Khersonsky and Tawfik, 2010; Gupta, 2016). This secondary function is called promiscuous, underground metabolism, or cross activity. In other words, the enzyme’s function, which has no physical relevance, can be classified as a promiscuous function due to the enzyme catalytic promiscuity, which deals with the ability of an enzyme active site to catalyze more than one chemical conversion. The broader implications of the “darker” aspect of enzyme promiscuity are largely ignored, and the idea of “one enzyme-one substrate-one reaction” prevails (Khersonsky and Tawfik, 2010). Poor catalytic efficiencies generally characterize enzymatic side activities, thus are likely to be coincidental and not the direct result of natural selection. The chemical mechanisms and evolvability of underground activities have received much attention at the level of individual enzymes (Notebaart et al., 2018).

Enzyme immobilization is a promising strategy for developing the efficiency of biocatalysts, and in the biomass industry, immobilized enzymes potentially improve the lignocellulose bioconversion and thus play an influential role in bioethanol production (Girelli et al., 2019, 2021; Norouzi et al., 2020; Ariaeenejad et al., 2020b; Motamedi et al., 2021a). In this regard, nano-carriers are promising conventional matrices for enzyme immobilization. In recent studies, magnetic nano cellulose carriers have been synthesized from sugar beet pulp (SBP), which has been used to immobilize enzymes cocktail. The results showed a significant increase in the yield of released fermentable sugars (Ariaeenejad et al., 2021c). In another study, cellulose nanocrystals were used to immobilize bifunctional biocatalysts extracted from the metagenome.

In that, improvement facilitating enzyme recycling and improved storage, thermal stability, and kinetic properties was observed (Ariaeenejad et al., 2021a).

In addition, several reports have been presented on improving enzymes’ efficiency and stability by immobilizing metagenome-derived cellulases and xylanases on different carriers (Ariaeenejad et al., 2019b, 2020a,c). Ultimately, this action increased the reducing sugars resulting from the degradation of lignocelluloses and ethanol production.

This study identified a novel recombinant bifunctional enzyme (xylanase/β-glucosidase) from the cattle rumen metagenome. Further enzymatic characterization of the PersiBGLXyn1 confirmed the multi-functional potentials and β-glucosidase underground activity. The bifunctional recombinant enzyme has the main activity of xylanase and a promiscuous activity of β -glucosidase for saccharification of CRW. The enzyme was physically immobilized on a nanocellulose carrier (NC) to enhance the underground activity. In our previous studies on the conjugation of the enzymes on to the NC carrier from SBP, the enzyme was immobilized *via* chemical conjugation onto the carrier surface using dopamine functionalization of NC (Ariaeenejad et al., 2021a,c). However, the existence of hydroxyl functional groups and NC carrier net negative charges could provide an effective physical enzyme immobilization on the NCs. So, herein, the performance of the synthesized pristine NC carrier was evaluated in the physical immobilization of PersiBGLXyn1. Afterward, the potential of the free and immobilized PersiBGLXyn1 in bioethanol production from the organic solvent pretreated CRW through the separate enzymatic hydrolysis and fermentation (SHF) process in optimal performance conditions to complete saccharification and fermentation was investigated. Enzymatic saccharification of agro-waste by bifunctional immobilized enzyme resulted in high production of fermentable sugars and ethanol, confirming the immobilization technique’s high efficacy.

## Materials and methods

### Materials

For the preparation of the nanocellulose carrier, sugar beet pulp (SBP) was provided by Sugar Beet Seed Institute (Karaj, Iran) and sulfuric acid (H_2_SO_4_, 98%) was purchased from Sigma–Aldrich. For enzyme assays, the 3, 5-dinitrosalicylic acid (DNS), bovine serum albumin (BSA), beechwood xylan, phenylmethane sulfonyl fluoride, imidazole, monosodium dihydrogen orthophosphate, metal ions, ethylene diaminetetra acetic acid (EDTA), urea, phenyl methyl sulfonyl fluoride (PMSF), sodium dodecyl sulfate (SDS), cetrimonium bromide (CTAB), tween 20, Triton X-100, Dithiothreitol (DTT), avicel, β-glucan, filter paper, locust bean gum (LBG), carboxymethyl cellulose (CMC) and β-pNPG were purchased from Sigma-Aldrich. The CRW was from the local market (Karaj, Iran). For the cloning, expression and purification of the PersiBGLXyn1, the T4 DNA ligase, *Nhe*I and *Sal*I restriction enzyme and Gel Extraction kit were purchased from Thermo Fisher Scientific (Waltham, United States) and kanamycin (Duchefa, Haarlem, The Netherlands), Isopropyl β-D-1-thiogalactopyranoside (IPTG, Sigma–Aldrich), the Luria-Bertani medium (LB broth, Merck, Germany) and Ni2 + -NTA Fast Start Kit (Qiagen, Hilden, Germany) were used.

### Mining of the bifunctional xylanase/β-glucosidase sequences

For mining enzymes with xylanase/β-glucosidase bifunctional activities the metagenomic data from the cattle rumen was used as described previously (Gharechahi et al., 2020). The raw data quality control, and metagenomic short reads assembly were performed, using FastQC and MEGAHIT, repectively. Assembled contigs were analyzed to identify putative enzymes. A sequence of computational steps was applied to select the appropriate xylanase enzyme with β-glucosidase underground activity as previously described (Ariaeenejad et al., 2021b), which included both sequence and structure based computations. First, the metagenomic sequences with high sequence similarity to selected xylanase enzymes with known activity were screened, and after that, the structural similarity of these enzymes with β-glucosidase enzymes was checked. The final selected enzyme was characterized experimentally.

### Bifunctional xylanase/β-glucosidase cloning, expression and purification

For polymerase chain reaction (PCR) amplification, metagenomic DNA template of cattle rumen from an earlier study was used (Gharechahi et al., 2020). Two pairs of primers, the forward primer (5′- TAATAGCATATG ATGAAGAAACTCATAATAGGTTTG-3′) with *NheI* restriction site and the reverse primers (5′- TGATAG GTCGAC TTATTTCACAACCAATGCCTTG-3′) with *SalI* restriction site were used for amplification. After extracting PCR product from agarose gel 1% (w/v) by the gel extraction kit, the purified DNA fragments were cloned into the pET28a as previously described (Ariaeenejad et al., 2019a; Motamedi et al. 2021b). Then, pET28a plasmid was transformed into *E. coli* BL-21 (DE3) cells and the resulting recombinant cell were cultivated at LB medium at 37°C. After incubation with IPTG, N-terminal Histidine-tagged recombinant enzyme was purified by utilizing Ni2 + -NTA Fast Start Kit. The purified PersiBGLXyn1 was evaluated by sodium dodecyl sulfate-polyacrylamide gel electrophoresis (SDS-PAGE). The nucleotide sequence of PersiBGLXyn1 was registered in the GenBank database with submission ID MT023060.

### Enzyme activity assay

Xylanase and β -glucosidase activities were quantified using beechwood xylan (1% w/v) and β -D-glucopyranoside ( β -pNPG) in the concentration of 10 mM as substrate, respectively. For xylanase activity detection, the released reducing sugar during enzymatic hydrolysis under pH 8.0 (phosphate buffer, 50 mM) at 60°C for 15 min was measured. The reduction of yellow-colored 3,5-dinitrosalicylic acid (DNS) to the orange-red-colored was investigated by recording the absorbance at 540 nm (Miller, 1959). The β -glucosidase activity of the PersiBGLXyn1 was assayed at 40°C in 50 mM phosphate buffer pH 8.0 for 10 min. By adding Na_2_CO_3_ (1 M) and reading the absorbance at 405 nm, the reaction was stopped (Parry et al., 2001). The amount of PersiBGLXyn1 that releases 1 μmol of xylose or pNP per minute, one unit of the enzyme activity was defined.

### Enzyme immobilization

#### Synthesis of nanocellulose carrier

Firstly, the fine powder of SBP was prepared by grinding its raw powder in a blender. For the enzymatic hydrolysis step, 2 g of SBP powder were dispersed in 100 ml of phosphate buffer (pH 8.0, 50 mM), which contained the enzymes cocktail (PersiCel2, PersiCel4, PersiBGL1, PersiXyn2 and PersiManXyn1; Ariaeenejad et al., 2021c). The enzyme cocktail was contained 0.5 mg/ml of cellulase and hemicellulase enzymes. After incubation at 80, for 3 days, the resultant enzyme-treated precipitates were washed with deionized water and dried. In the next step, 1 g of the dried enzyme-treated SBP was dispersed in 40 ml of sulfuric acid solution (60%), and stirred at 50°C, for 4 h. Then, the mixture was centrifuged and the collected precipitates were continuously washed using copious water until the pH of the resultant suspension was reached 5. Finally, the precipitates were dried at 80°C in the oven (Ariaeenejad et al., 2021a,c).

#### Characterization of NC-carrier

Transmission electron microscopy (TEM, TEM Philips EM 208S, 100 kv), and field emission scanning electron microscopy (FESEM, TESCAN MIRA II microscope; 20 kV) have been exploited to characterize the nano-carrier.

For FESEM analysis, the sample powders were coated by a thin gold layer before SEM observations. For TEM analysis, the sample’s powders were dispersed in ethanol (1 mg/ml), using an ultrasonic equipment. One drop of the suspensions was placed on carbon film-covered copper grids (400 mesh). After drying the grid, TEM microscopy was conducted.

#### Enzyme immobilization on NC-carrier and efficiency of the immobilization

PersiBGLXyn1 were immobilized on NC-carriers, by dispersing the different amounts of NC-carrier in the enzyme solution. Firstly, 30 μ L persiBGLXyl1 solution (0.5 mg/ml = 2 U/mg) in phosphate buffer (pH 8.0, 50 mM), was incubated at 50 °C for 4 h. Then the precipices were collected using a centrifuge, and washed with the 50 mM phosphate buffer, and then dried at room temperature and stored at 4°C for further use.

Immobilization efficiencies by measuring the initial and final concentrations of enzyme were obtained (Marshall and Williams, 1993). The supernatant was separated from the immobilization medium by centrifugation 5,000 rpm for 5 min and the enzyme concentration was calculated according to the Bradford assay.

The percentage of immobilization efficacy was determined using Eq. (1) and through the differences between the initial (C_i_) and the final concentration (C_s_) of PersiBGLXyn1 in the immobilization.


(1) $$\mathrm{Immobilization efficacy (}\%)=\frac{Ci-Cs}{Cs}\times 100$$

The relative activities of immobilized enzyme was estimated by Eq. (2):


(2) $$\mathrm{Relative activity}\;\left(\%\right)=\left(\frac{A}{A0}\right)\times 100$$

Where A is the activity of the immobilized and A_0_ is the activity of free enzyme.

For the reusability of the immobilized PersiBGLXyn1, its residual activity was measured after reusing in the reaction cycles (at 50°C, pH = 8, incubation time of 1 h). The first cycle measured activity was defined as 100% and during each cycle, the immobilized enzyme was collected after incubation with the substrate. Again, after washed with phosphate buffer, it was added to the fresh substrate solution.

### The stability of free PersiBGLXyn1

The stability of the enzyme against harsh conditions (wide range of pH, temperatures and inhibitors) was investigated using the following experiments.

#### Cations, inhibitors and chemicals influence

The effect of metal ions on the enzyme activity was investigated by adding MgCl_2_, CaCl_2_, NaCl, MnCl_2_, CuSO_4_, FeSO_4_, ZnCl_2_, CdSO_4_, NiSO_4_, CoSO_4_, PbNO_3_, and KCl to the reaction mixture. The influence of ionic (SDS) and nonionic surfactants (Tween 20 and Triton X-100) in the concentration of 1% and inhibitors EDTA, Urea, PMSF, NaN_3_, DTT at 10 mM concentration and ethanol and methanol (10% v/v) were also examined. Each compound with enzyme was pre-incubated for 30 min at room temperature. The enzymatic activity in the absence of chemicals was taken as control (100% activity) and relative activities were calculated.

#### Thermal and pH stability

The thermostability of the enzyme was evaluated by incubating the PersiBGLXyn1 at ranging temperatures 30 to 80°C in the absence of substrate and determining its relative activity after 120 min.

For investigating the pH stability, the relative enzyme activity was examined after enzyme was affected a wide range of pH from 4.0 to 9.0 in the absence of substrate for 120 min. The sodium citrate buffer (pH 4.0 to 5.0), potassium phosphate buffer (pH 6.0 to 8.0) and Tris-HCL buffer (pH 9.0) was used for different pH.

### Characterization of free and immobilized PersiBGLXyn1

#### Catalytic activity under various conditions of pH and temperature

The optimum temperature, at which the maximum activity of the enzyme occurred, were carried out by analyzing the free and immobilized enzyme activity using xylan (1% w/v) and β-pNPG (10 mM) as substrates in temperatures varying between 30 to 80°C, during 10 min incubation of the reaction mixture at each temperature. The optimum pH for the free and immobilized enzyme was similarly assayed by investigating enzyme activity in the range of 4.0 to 9.0 at optimum temperature (60°C and 40°C for xylanase and β -glucosidase activities). The optimal reaction pH was assessed using several buffers with varying pH values, including sodium citrate buffer (50 mM, pH 4.0 to 5.0), potassium phosphate buffer (50 mM, pH 6.0 to 8.0), and Tris–HCL buffer (50 mM, pH 9.0).

#### Thermal denaturation

To evaluate the thermal denaturation of the free and immobilized PersiBGLXyn1, half-life (t½) and decimal reduction time (D-value), were calculated which tells the influence of temperature on enzyme denaturation. The half-life (t_1/2_) was determined by measuring activity assay and xylanase activity was incubated at 70 °C and 80 °C intervals of 0, 60, 120, 180, and 240 min. Also β -glucosidase activity was incubated at 70 °C and 80 °C for 20 min at time intervals of 0, 5, 10, 15, and 20 min, in the absence of substrates. The enzymatic activity at 0 min was taken as 100% and results were expressed as enzyme half-life (t_1/2_). A graph of-Ln A/A_0_ at various periods versus time (min) was plotted and its slop was determined as the inactivation rate constant (k_d_) according to the below equation:


(3) $$Ln\frac{A}{A_{0}}=k_{d}t$$

Half-life and D-value of the enzyme were described as the time required to lose 50 and 90% of initial xylanase and β-glucosidase activity, respectively. For calculating them using the Eqs. (4) and (5) after calculation of the first-order denaturation constant (K_d_; Ariaeenejad et al., 2021b).


(Eq 4) $$t_{1/2}=\frac{Ln2}{K_{d}}$$

(Eq 5) $$D=\frac{Ln10}{K_{d}}$$

#### Kinetic characterization

The Michaelis–Menten curves were built to investigate the kinetic parameters of the free and immobilized PersiBGLXyn1 using xylan (0.25 to 5% w/v) and β-pNPG (0.5 to 10 mM) at pH 8.0. The K_m_ and V_max_ of enzymes were estimated based on the results.

### Effect of free and immobilized PersiBGLXyn1 on the degradation of CRW

#### Organic solvent pretreatment of CRW

The CRW used in this study was analyzed for its components, including holocellulose (consisting of cellulose and hemicellulose), lignin, extractive, and ash, based on standard methods (Liao et al., 2017). Pretreatment of CRW was performed as described in the former study (Ravindran et al., 2017). The CRW (25 g) was soaked in 250 ml of ethanol 50% (v/v) followed by the addition of sulphuric acid 1% (w/w) as catalyst. The reaction was carried out at 120°C for 30 min. After cooling at room temperature, the solution was filtered and washed, followed by centrifugation to obtain the precipitated solid. Finally, the solid fraction was dried and stored for further analysis.

#### Enzymatic hydrolysis of CRW

For enzymatic hydrolysis, the reaction mixture containing the dried CRW (140 mg/ml) and the free and immobilized PersiBGLXyn1 (0.037 U/g biomass) under pH 8.0 were incubated at 50°C for 10 days. Every 48 h, the samples were taken and the released reducing sugar was measured by DNS method.

### Ethanol fermentation of hydrolyzed CRW

The hydrolysates from the pretreated CRW were subjected to the fermentation process. The fermentative microorganism *Saccharomyces cerevisiae* was activated in the medium containing 0.75 g yeast extract, 0.75 g malt extract, 1.25 g soy peptone, and 1.25 g glucose. The fermentation experiment was performed using 5% (w/v) of CRW and 10 ml culture (pH 6.8) followed by incubation at 180 rpm, 28°C for 72 h. Then, mixtures were centrifuge at 8000 rpm for 5 min and supernatants were collected for measuring the ethanol content.

After the reaction, the ethanol content was measured using the potassium dichromate oxidation method (Caputi et al., 1968). Briefly, 100 μl of the potassium dichromate reagent (3.4 g of K_2_Cr_2_O_7_ in 50 ml distilled water and 32.5 ml of H_2_SO_4_ in the total volume of 100 ml) was added to the 100 μl of sample and incubated for 10 min at 60°C. Afterward, the absorbance was recorded at 584 nm. The ethanol content was estimated using the standard curve representing the relationship between the ethanol concentration and absorbance at 584 nm.

### Statistical analysis

All experiments in this study were performed in three replications and the mean and standard deviation values were calculated using software Microsoft Office Excel 2016.

## Results and discussion

### Mining of the bifunctional xylanase/β-glucosidase sequence

After performing a set of computational steps on the raw data (Ariaeenejad et al., 2021b), a metagenomic sequence, PersiBGLXyn1, was selected for experimental characterization. PersiBGLXyn1 was structurally similar to bacterial xylanase from *Globitermes brachycerastes* with PDB code 4HU8 and a bacterial β-glucosidase from *Halothermothrix orenii* with PDB code 3TA9 with 100% confidence, suggesting the bifunctional activity of PersiBGLXyn1.

### PersiBGLXyn1 cloning, expression and purification

After amplifying the sequence of PersiBGLXyn1 from the metagenomic DNA, it was cloned in pET-28a vector and overexpressed in *E. coli* BL21 (DE3). The recombinant enzyme with N-terminus His-tag was then purified using Ni-NTA Fast Start Kit. As shown in the SDS-PAGE gel, the single bands for PersiBGLXyn1 were confirmed by a molecular weight corresponding to the 42.23 kDa.

### Characterization of the freePersiBGLXyn11

The activity and stability of enzymes against metal ions and inhibitors are essential for the enzymatic biodegradation of lignocellulosic materials and, finally, ethanol production. The effect of metal ions, chemical modulators, and surfactants on the activity of PersiBGLXyn1 was examined by calculating the relative β-glucosidase and xylanase activities (Table 1). The enzyme showed a high ability to withstand most tested metal ions and inhibitors. Among the metal ions, PbNO_3_ and ZnCl_2_ stimulated the β-glucosidase activity to 125.20 and 121.14%, while CdSO_4_ and CaCl_2_ dramatically inhibited the β-glucosidase activity. When the enzyme was incubated with MnCl_2_ and FeSO_4_, and CaCl_2_, maximum xylanase activities were observed and showed 147.66, 121.94, and 121.92%, respectively. The effects of monovalent and divalent metal ions on enzymatic activity may positively affect the catalytic rate due to their roles in connecting the enzyme and substrate and in the protein structural alterations. The inhibitory effects of Ca^2+^, Cd^2+^and Cu^2+^ have been reported for β-glucosidases from rumen metagenome, *Bacillus halodurans* and *Dictyoglomus turgidum* (Xu et al., 2011; Limauro et al., 2018; Ariaeenejad et al., 2020d).

**Table 1:** Effect of metal ions, chemical agents and detergents on β-glucosidase and xylanase activities of the PersiXynBGL1.

	Relative β-glucosidase activity (%)	Relative Xylanase activity (%)
**Metal ions**		
Control	100	100
MgCl_2_	105.30	116.08
CaCl_2_	10.96	121.92
NaCl	91.86	120.34
MnCl_2_	100.13	147.66
CuSO_4_	102.17	92.11
FeSO_4_	108.13	121.94
ZnCl_2_	121.14	92.42
CuSO_4_	46.23	92.11
CdSO_4_	18.45	96.84
NiSO_4_	104.17	79.65
CoSO_4_	104.80	101.10
PbNO_3_	125.20	86.43
KCl	95.64	75.39
**Surfactants**		
SDS	90.79	69.87
CTAB	106.28	82.64
Tween 20	103.90	75.07
Triton X-100	132.38	157.72
**Inhibitors**		
EDTA	76.28	85.16
Urea	87.82	97.03
PMSF	100.49	79.17
NaN_3_	93.98	98.16
DTT	99.91	133.59
Ethanol	86.79	107.09
Methanol	94.88	118.13

The effect of surfactants and inhibitors on PersiBGLXyn1 activity was also studied (Table 1). CTAB and Tween 20 were found to increase the β-glucosidase PersiBGLXyn1 activity. The xylanase PersiBGLXyn1 activity was decreased in the presence of these surfactants. Remarkably, EDTA had a small negative effect on both β-glucosidase and xylanase activities and reduced their activity to 76.28 and 85.16%, respectively. In addition, there was no remarkable alteration in the β-glucosidase activity of PersiBGLXyn1 in the presence of strong enzyme inhibitors, including DTT and PMSF, which act on the-SH groups and the seryl hydroxyl group of the enzyme (Mehta and Satyanarayana, 2016). In contract, the xylanase PersiBGLXyn1 activity increased to 133.59% after the addition of DTT and dropped to 79.17% when the enzyme was incubated with PMSF. Activation of xylanase activity by DTT was reported before by xylanase from *Aspergillus niger* (Romanowska et al., 2006). The relationship between the reduced form of cysteine residues and xylanolytic activity may be the cause (Heinen et al., 2018). In addition, the stability of the enzyme was enhanced by organic solvents, ethanol, and methanol. The activity of the PersiBGLXyn1 in the presence of ethanol, methanol, SDS, and EDTA was higher than the hyper thermostable bifunctional cellobiohydrolase-xylanase from the *Chaetomium thermophilum* and bifunctional xylanase/endoglucanase enzyme from *Streptomyces thermocerradoensis* (Gama et al., 2020; Han et al., 2020).

**Figure 1:**
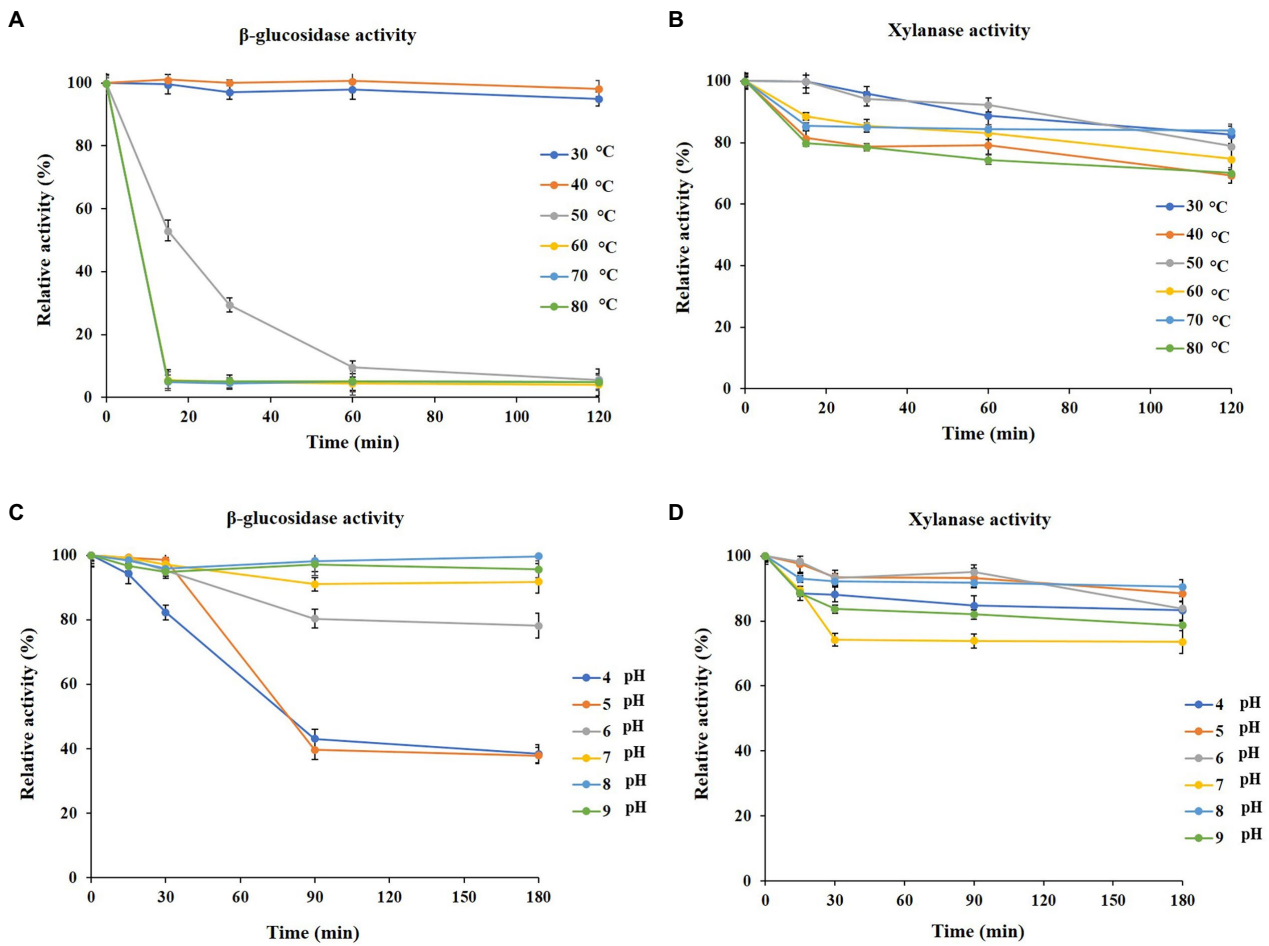
Properties of purified PersiBGLXyn1. **(A)** Effect of temperature on the β-glucosidase stability after 120 min of pre-incubation at 30 to 80°C. **(B)** Effect of temperature on the xylanase stability after 120 min of pre-incubation at 30 to 80°C. **(C)** Effect of pH on β-glucosida stability after 180 min of pre-incubation at pH 4 to 9. **(D)** Effect of pH on xylanase stability after 180 min of pre-incubation at pH 4 to 9.

To analyze the thermal stability and pH stability of PersiBGLXyn1, xylanase and β-glucosidase activities were measured at a wide temperature and pH range. PersiBGLXyn1 β-glucosidase activity decreased at temperatures above 40°C, and this experiment showed that the enzyme loses its β-glucosidase activity after 15 min of incubation at 60°C to 80°C (Figure 1A). The PersiBGLXyn1 xylanase activity was thermostable since it retained over 70% of its initial activity at 80°C after 120 min (Figure 1B).

Additionally, the pH stability of PersiBGLXyn1 was assayed at a wide range of pHs and various incubation times. According to the results, Figure 1B, the enzyme could retain most of its xylanase activity compared with β-glucosidase activity at pH from 4.0 to 9.0. In acidic conditions (pH 4.0), 83.26% xylanase activity was retained after 180 min, and the enzyme could maintain more than 78.62% of its xylanase activity at high alkaline conditions (pH 9.0; Figure 1C). On the other hand, the β-glucosidase activity of the enzyme at pH 4.0 and 9.0 were 38.43 and 95.63% after 180 min incubation (Figure 1D).

Enzymes with a wide pH and temperature range activity and high resistance against harsh conditions are favorable for largescale industrial reactions for prolonged durations. Thermostable enzymes with β-glucosidase and xylanase activities can improve the bioconversion of lignocellulosic biomass during the cost-effective process. Compared to the other bifunctional enzymes with xylanase activity, it can be noted that a chimeric bifunctional enzyme (GA_2(syn_SKYAP01))exhibited less than 60% xylanase activity after incubation at 60°C for 120 min (Purohit et al., 2021). Furthermore, the xylanase activity of the PersiBGLXyn1 at high temperature was found to be more stable than previously reported bifunctional enzymes. A purified bifunctional xylanase/endoglucanase (RuCelA) from yak rumen metagenome was reported to become, completely inactivated after 60 min incubation at 70°C (Chang et al., 2011). In addition, the xylanase activity of a bifunctional xylanase/cellulase (Xyn-CBM-Cel) from camel rumen decreased up to 64% after 1 h at 50°C (Khalili Ghadikolaei et al., 2017). In another report, it was shown that the yak rumen’s bifunctional enzyme β-glucosidase/xylosidase (RuBG3B) was inactivated after 20 min of incubation at 50°C (Bao et al., 2012).

Also, pH stability studies of the xylanase from a rumen metagenomic library showed lower stability than PersiBGLXyn1 both at acidic and alkaline solutions with less than 30 and 80% activities at pH 4.0 and pH 9.0 after 60 min, respectively (Zhou et al., 2020). The results obtained in our study gave a higher value of the β-glucosidase pH stability in both acidic and alkaline conditions than PersiBGLXyn1 when compared with a thermostable β-glucosidase (DturβGlu) from *Dictyoglomus turgidum* showing less than 40 and 60% activities at pH 4.0 and pH 9.0, respectively (Limauro et al., 2018). A wide range of thermal stability and high pH tolerance of the PersiBGLXyn1 in a wide range is desirable for various industrial applications especially for enzymatic saccharification of the biomass and second-generation ethanol production.

### Characterization of NC-carrier

**Figure 2:**
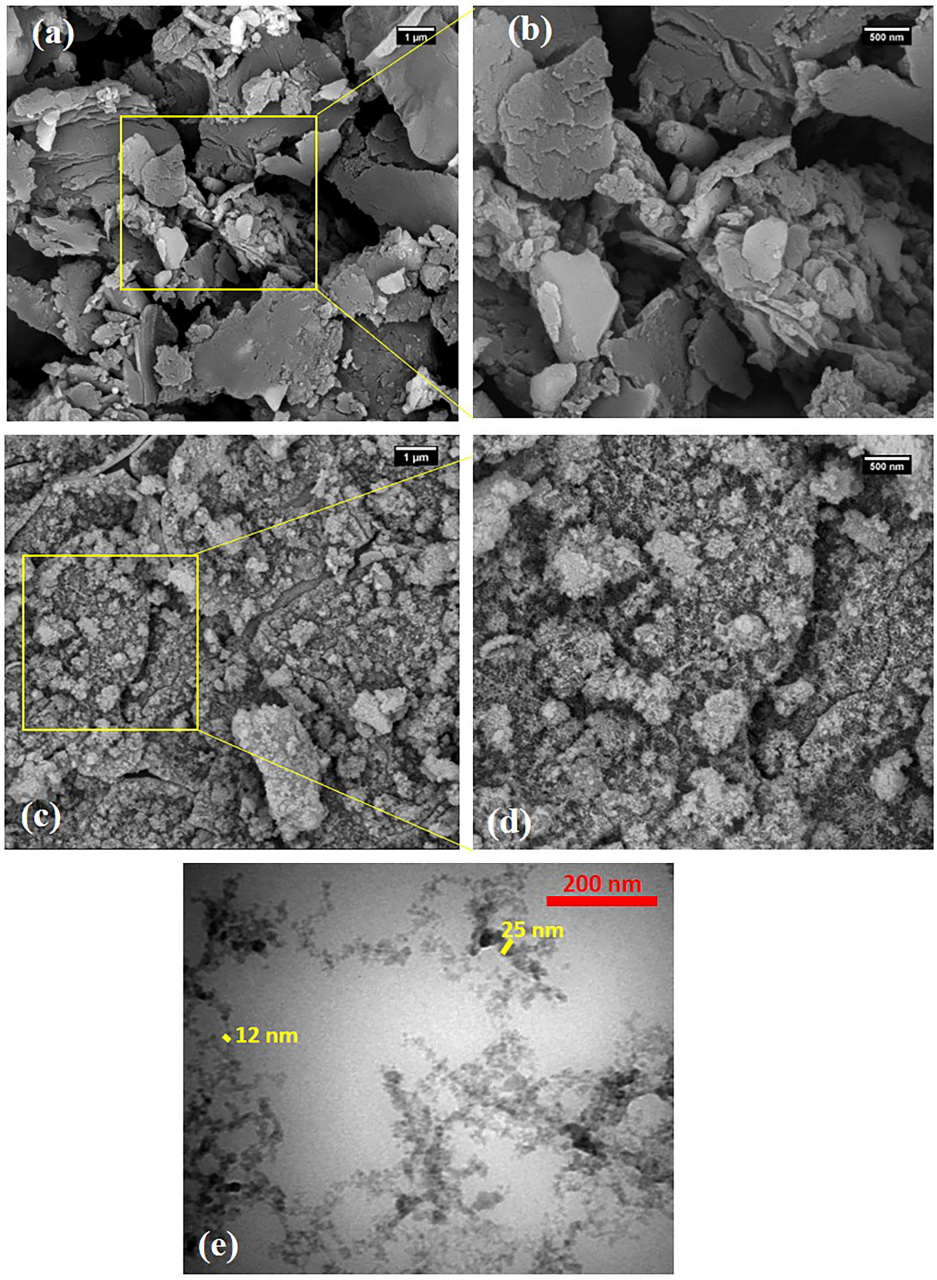
SEM images of **(A,B)** raw SBP powder and **(C,D)** NC carrier in different magnifications and **(E)** TEM image of NC carrier. Magnification of figures were a,c-20,000X, b,d-50,000X, and e.40000X.

SEM images of raw SBP powder and as-synthesized NC-carrier at different magnifications were displayed in Figure 2. The SEM images of SBP indicated several intact hunks with wide micrometric size ranges, that some of them were stacked together. Conversely, the NC sample images displayed the dense and aggregated acicular particles with diameter in a range of 15 to 25 nm and average length of 40–150 nm. This may be due to the fact that NC particles tend to self-assembling during the drying process which hardened identification of individual NCs using FESEM images (Yahya et al., 2018). Consequently, TEM analysis was used to investigate the dimension/morphology of isolated particles in NC sample (Figures 2E,F). The results confirmed that the hydrolyzed NC particles were assembled fibrillary and made aggregated fibers with an average diameter of 10–25 nm.

### Properties of the free and immobilized PersiBGLXyn1

**Figure 3:**
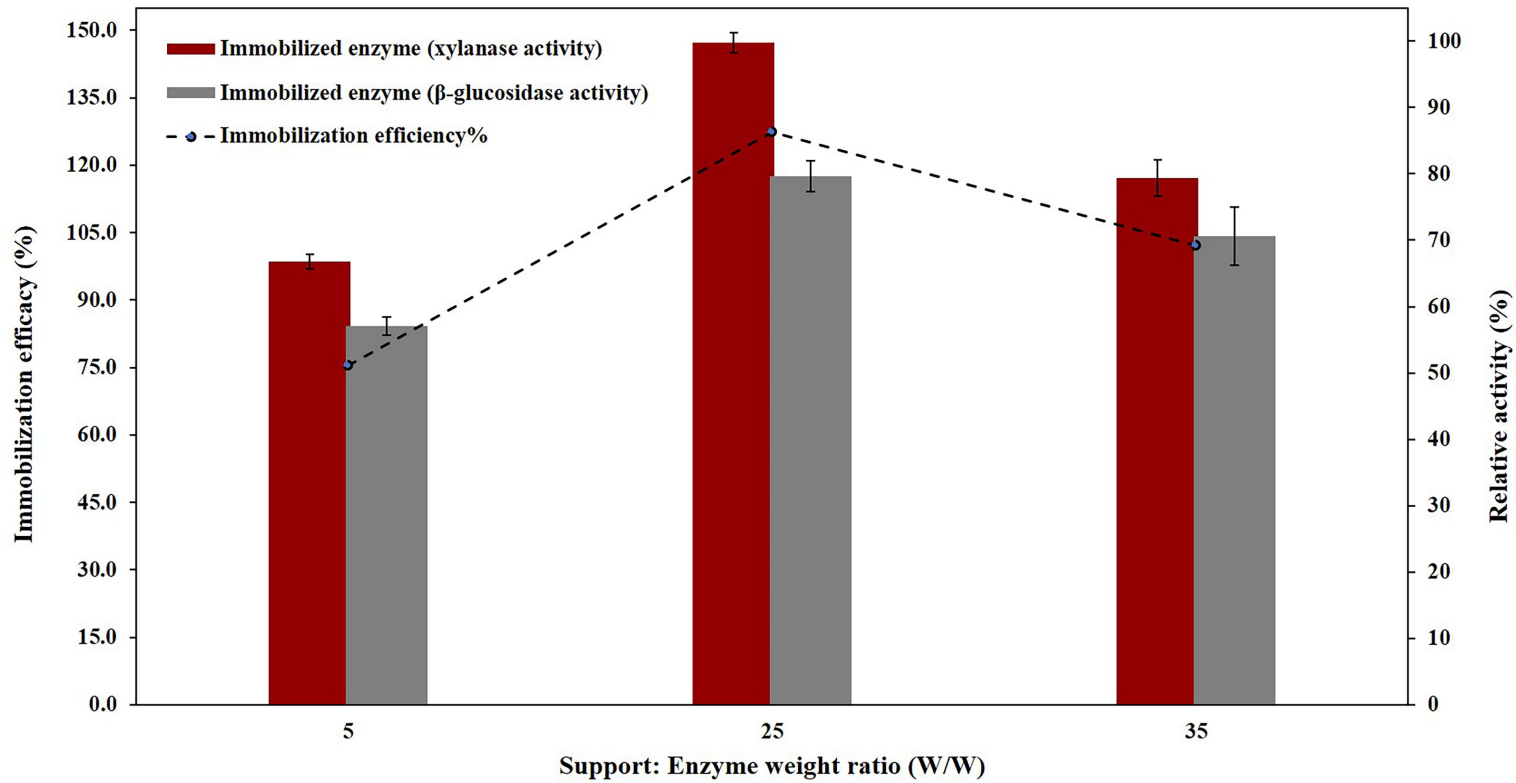
PersiBGLXyn1 relative activity (%) after immobilization on NC nano-carrier at various support:Enzyme weight ratios.

In order to evaluate the best weight ratio of the NC-carrier and enzyme immobilization efficacies and relative activities against NC-carrier:enzyme weight ratios were calculated for the immobilized PersiBGLXyn1.The results showed that with remained constant concentration of enzyme and when increasing the amounts of NC-carrier:enzyme weight ratios, both immobilization efficiency and relative activity were increased. Until, their maximum levels of them observed at weight ratio of 25, and then decreased afterward (Figure 3). Therefore, the ratio of support:enzyme of 25, was selected as the best ratio for PersiBGLXyn1 immobilization and utilized for further enzyme assays. Moreover, it could be seen that upon immobilization on NC-carrier, the xylanase activity was improved better than that of β-glucosidase activity, at all examined ratios. For instant, the ratio of support:enzyme of 25, the relative activity of xylanase and β-glucosidase in immobilized enzyme was enhanced up to 147.22 and 117.51% respectively, compared to free enzyme.

**Figure 4:**
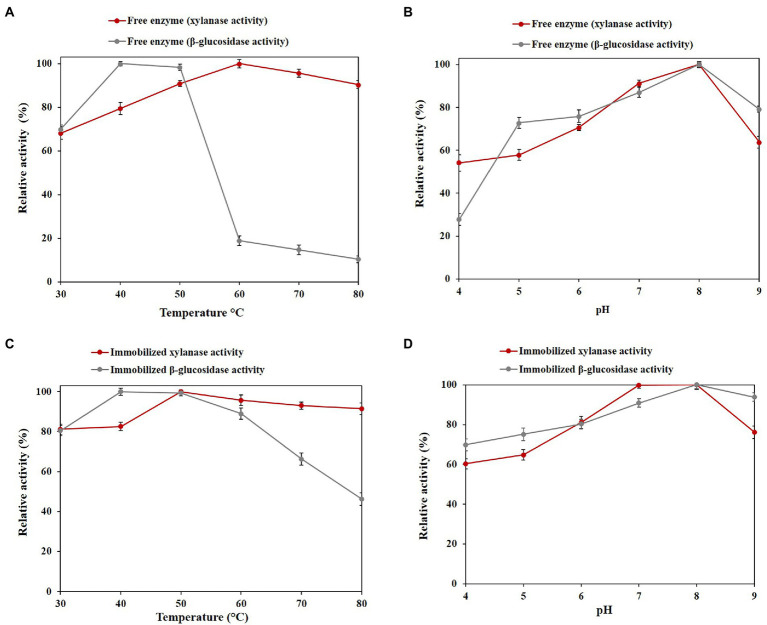
Optimal values of free and immobilized PersiBGLXyn1. **(A)** Effect of temperature on the xylanase and β-glucosidase activity of free enzyme at 30 to 80°C. **(B)** Effect of pH on the xylanase β-glucosidase activity of free enzyme after at pH 4 to 9. **(C)** Effect of temperature on the xylanase and β-glucosidase activity of immobilized enzyme at 30 to 80°C. **(D)** Effect of pH on the xylanase and β-glucosidase activity of immobilized enzyme at pH 4 to 9.

The optimum temperature of the free enzyme was found to be at 60°C for xylanase activity and 40°C for β-glucosidase activity (Figure 4A). The optimum temperature of the immobilized enzyme is similar to the free enzyme, with the difference that after the optimum temperature, with increasing temperature, both functions significantly increase activity (Figure 4C).

Regarding the highest xylanase and β-glucosidase activities, both the free and immobilized PersiBGLXyn1 showed a similar optimal pH of 8.0, indicating the immobilization process did not alter the pH optima (Figures 4B,D).

Metagenome-derived bifunctional cellulase/xylanase (PersiCelXyn1) from cow rumen microbiota has shown promising activity in a wide range of temperature and pH. This study showed that bifunctional enzymes with high activity at a wide temperature and pH range are beneficial to overcome the lignocellulosic biomass resistance structure (Ariaeenejad et al., 2021b).

In this study, the PersiBGLXyn1 retained 90.46% of its xylanase activity at 80°C and a decline in relative β-glucosidase activity was observed at the high temperatures. It is clear that immobilization of the enzyme provided more enzymatic activity than free one and resulted in a remarkable increase of β-glucosidase activity up to 46.30% at 80°C (Figure 4A). Increasing the enzymatic activity, especially the β-glucosidase activity of the PersiBGLXyn1 at higher temperatures, may be due to the formation of covalent bonds between the enzyme and the surfaces of support which improved the molecular structure rigidity of the PersiBGLXyn1 (Ariaeenejad et al., 2020e). Similarly, immobilization of a novel metagenome-derived bifunctional mannanase/xylanase (PersiManXyn1) on cellulose nanocrystals enhanced enzyme activity and improved the conversion of the lignocellulosic biomass for utilization of the enzyme in industrial applications (Ariaeenejad et al., 2021a). Likewise, immobilizing the cellulase and hemicellulose enzyme cocktail improved the thermal stability optimum temperature and elevated the yield of released reducing sugars (Ariaeenejad et al., 2021c). In another study, immobilization of β-glucosidase on magnetic MnO2 enhanced the enzyme’s thermal stability and optimum pH (Huang et al., 2021). Enzyme immobilization of xylanase within glutaraldehyde-activated calcium alginate beads improved the storage stability and increased optimum pH and temperature (Kumar et al., 2017). In addition, the immobilized PersiBGLXyn1 was more active than the free enzyme at all tested pH and kept 60.32 and 76.19% of its xylanase activity at the highest acidic (pH 4.0) and alkaline (pH 9.0) conditions. While, the free PersiBGLXyn1 retained only 54.13 and 63.78% of its xylanase activity at pH 4.0 and 9.0. However, for β-glucosidase activity, the free enzyme showed only 27.72% of its maximum value at pH 4.0 which strongly increased to 69.81% in immobilized PersiBGLXyn1. At high alkaline conditions (pH 9.0.), the immobilization enhanced the β-glucosidase activity from 79.23% in free enzyme to 93.86%. These results confirmed the efficacy of the immobilization technique to improve the enzymatic activity of the PersiBGLXyn1, which is essential for future use in various biotechnological processes, especially in hydrolysis lignocellulosic biomass. Upon immobilization, a protective environment and a more rigid external support were provided, enhancing the enzymatic activity of bifunctional PersiBGLXyn1 and increasing the enzyme utility for industrial processes. Using the Lineweaver-Burk plot, the kinetic parameters of the PersiBGLXyn1 were estimated. The K_m_ and V_max_ for xylanase activity of the free enzyme were calculated 1.37 mg·mL^−1^, 697.91 U.mg^−1^ and these values were 4.44 mg·mL^−1^, 56.60 U.mg^−1^ for the β-glucosidase activity. Moreover, 0.75 and 0.97 mg·mL^−1^ were the K_m_ values calculated for the xylanase and β-glucosidase activities of the immobilized enzyme, respectively. Besides, the V_max_ of the immobilized PersiBGLXyn1 were 805.17 and 550.46 U.mg^−1^ for the xylanase and β-glucosidase activities, respectively. The V_max_ for both xylanase and β-glucosidase activity of immobilized PersiBGLXyn1 was higher than that of the free enzyme, while the xylanase and β-glucosidase K_m_ values of immobilized PersiBGLXyn1 was lower as compared to free enzyme. These results showed the higher affinity of an enzyme toward the corresponding substrate and demonstrated the efficiency of the immobilization to improve the catalytic activity (Kharazmi et al., 2020). An immobilized bifunctional xylanase/cellulase enzyme from *Pichia pastoris* exhibited the higher K_m_ and lower V_max_ for xylanase activity compared with the xylanase activity of the immobilized PersiBGLXyn1 (Liu et al., 2015). Another study reported the V_max_ of 0.525 U.mg^−1^ for the immobilized thermostable xylanase from camel rumen metagenome which was lower than the xylanase activity of the immobilized PersiBGLXyn1(Ariaeenejad et al., 2020f). Moreover, the immobilized PersiBGLXyn11 showed lower K_m_ and higher V_max_ for xylanase activity compared to immobilized xylanase isolated from *Bacillus licheniformis* (Irfan et al., 2020). It was also reported earlier that an immobilized β-Glucosidase from almond showed V_max_ of 41 U.mg^−1^ (Califano et al., 2018) and a β-glucosidase from *Aspergillus niger* had V_max_ of 0.108 U.mg^−1^ which were lower than the β-glucosidase activity of the immobilized PersiBGLXyn1 (Vazquez-Ortega et al., 2018). The stability of enzymes under elevated temperatures is important for bioethanol production. The advantages associated with working at high temperatures for ethanol production are a decrease in contamination, more accessible process design, and product development (Taylor et al., 2009). In this regard, the thermal denaturation of free and immobilized PersiBGLXyn1 was investigated at high temperatures and half-lives were calculated and presented in Table 2. The half-lives of xylanase and β-glucosidase activities of the enzyme prolonged remarkably after immobilization indicating improved stability of PersiBGLXyn1 at higher temperatures. Xylanase activity of free enzyme was more thermostable than β-glucosidase activity and exhibited lower K_d_ values. After immobilization, these values were reduced showing higher thermal stability of enzyme upon immobilization. Compared with immobilized enzyme, the xylanase activity of free enzyme demonstrated half-life of 216.60 min and 126.02 min at 70°C and 80°C, respectively. Whereas, the calculated t ½ of the xylanase activity of immobilized PersiBGLXyn1 was found to be 364.81 min at 70°C which declined to 223.59 min at 80°C. In a same study, a hyperthermostable xylanase from *Thermotoga maritima* exhibited half-life of 130 min after incubation at 100°C confirming the high tolerance of the xylanase at extreme temperatures (Yu et al., 2016). Additionally, the half-lives of the free enzyme β-glucosidase activity was much lower than its xylanase activity and showed the loss of 50% enzymatic activity after 3 to 4 min incubation at both temperatures. This result exhibits the sensitivity of enzyme β-glucosidase activity to extreme temperatures. In contrast, immobilization of the enzyme promotes its thermal stability, particularly its underground β-glucosidase activity. As illustrated in Table 2, the half-life of immobilized PersiBGLXyn1 β-glucosidase activity was 12 to 13 times higher than those of the free form at 70°C and 80°C. This can be explained by the effective role of nanocarrier that could protect the structure of enzyme against heat denaturation for addition time (Kharazmi et al., 2020). The same trend was observed for the results of D-values and indicated the resistance of the enzyme to heat denaturation, after immobilization. Results are in agreement with previous studies that reported higher half-lives of xylanase and β-glucosidase enzymes after immobilization on alginate beads and magnetic nanoparticles (Jampala et al., 2017; Huang et al., 2021). These findings demonstrated enhanced thermal stability of the PersiBGLXyn1 immobilized on NC carriers to be used as an effective biocatalyst for biomass hydrolysis.

**Table 2:** K_d_, half-life and D-value of xylanase and β-glucosidase activities of free and immobilized PersiXynBGL1.

Temperature (°C)	Free enzyme (xylanase activity)			Immobilized enzyme (xylanase activity)		
	K_d_ (min^−1^)	Half-life (min)	D-value (min)	K_d_ (min^−1^)	Half-life (min)	D-value (min)
80	0.0032	216.60	719.55	0.0019	364.81	1211.88
90	0.0055	126.02	418.65	0.0031	223.59	742.76
**Temperature (°C)**	**Free enzyme (β-glucosidase activity)**			**Immobilized enzyme (β-glucosidase activity)**		
	**K_d_ (min^−1^)**	**Half-life (min)**	**D-value (min)**	**K_d_ (min^−1^)**	**Half-life (min)**	**D-value (min)**
60	0.1683	4.11	13.68	0.0137	50.59	168.07
70	0.2058	3.36	11.18	0.0159	43.59	144.81

### Reusability studies of immobilized PersiBGLXyn1

**Figure 5:**
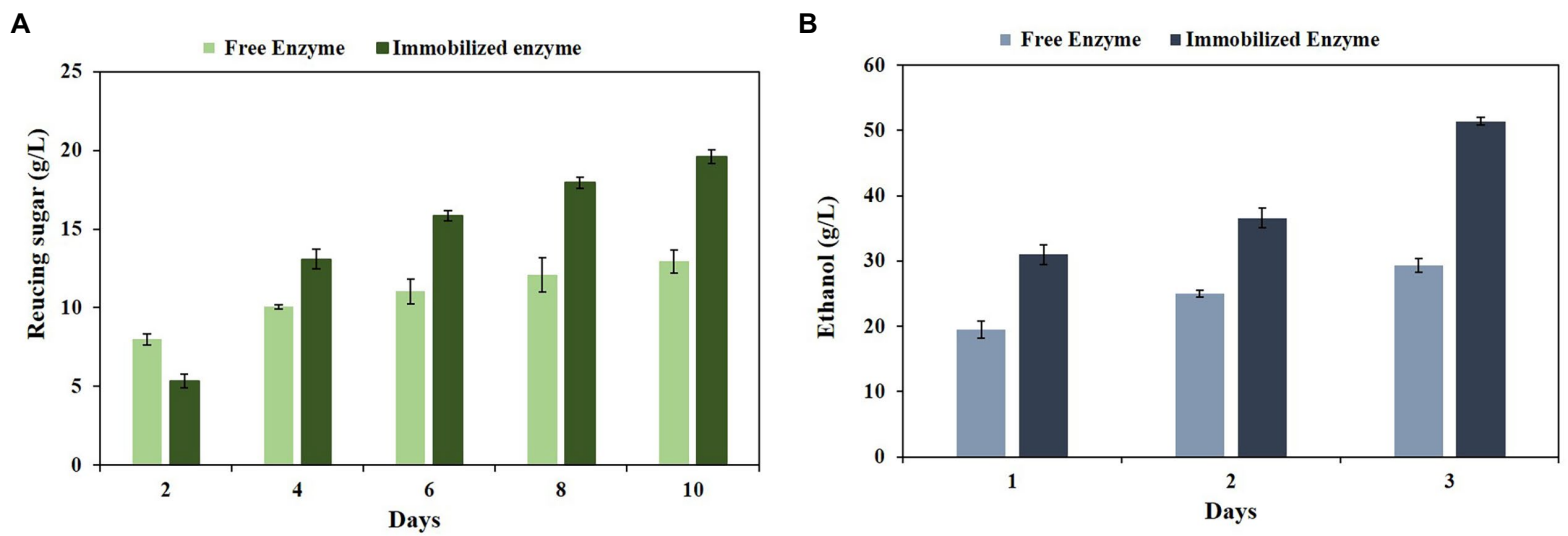
Reusability of PersiBGLXyn1 immobilized on nanocellulose (NC) carrier after 8 cycles.

The enzyme’s frequent uses are fundamental to the decline in the cost of processing at an industrial scale. Here, we examined the reuse of immobilized PersiBGLXyn1 through 8 cycles. As depicted in Figure 5, xylanase activity of the immobilized enzyme displayed 79.01% on its second use and this value reduced gradually during the subsequent cycles. Repeating use of immobilized enzyme over six cycles showed 56.11% xylanase activity. This value remained constant during the next cycles and reached 53.26% after final cycle. Similarly, there was a small decrease in relative β-glucosidase activity of immobilized PersiBGLXyn1 during repeated use. The β-glucosidase activity of enzyme was found to be 72.76% after second use and the enzyme retained 39.32% of its β-glucosidase activity after 8 cycles. It was noted that the xylanase activity of immobilized PersiBGLXyn1 after 8 cycles was higher than that of xylanase immobilized on modified superparamagnetic graphene oxide nanocomposite which showed less than 20% activity after 8 use (Mehnati-najafabadi et al., 2017). Likewise, alginate entrapped xylanase exhibited less than 40% activity when it was reused for 8 cycles, while the xylanase activity of PersiBGLXyn1 was more than 50% after being used for 8 cycles (Kumar et al., 2017). Additionally, compared with β-glucosidase activity of PersiBGLXyn1, the β-glucosidase immobilized on alginate–chitin and chitosan–chitin supports retained near 20% of its initial activity after 8 repeated use (Romo-Sánchez et al., 2014). In another work, recombinant β-glucosidase immobilized in Zeolite demonstrated less than 20% activity after 6 cycles (Ramírez-Ramírez et al., 2022). The obtained results indicated the effectiveness of PersiBGLXyn1 immobilization for improving its reusability and applications in different fields.

### Degradation of CRW By free and immobilized PersiBGLXyn1

**Figure 6:**
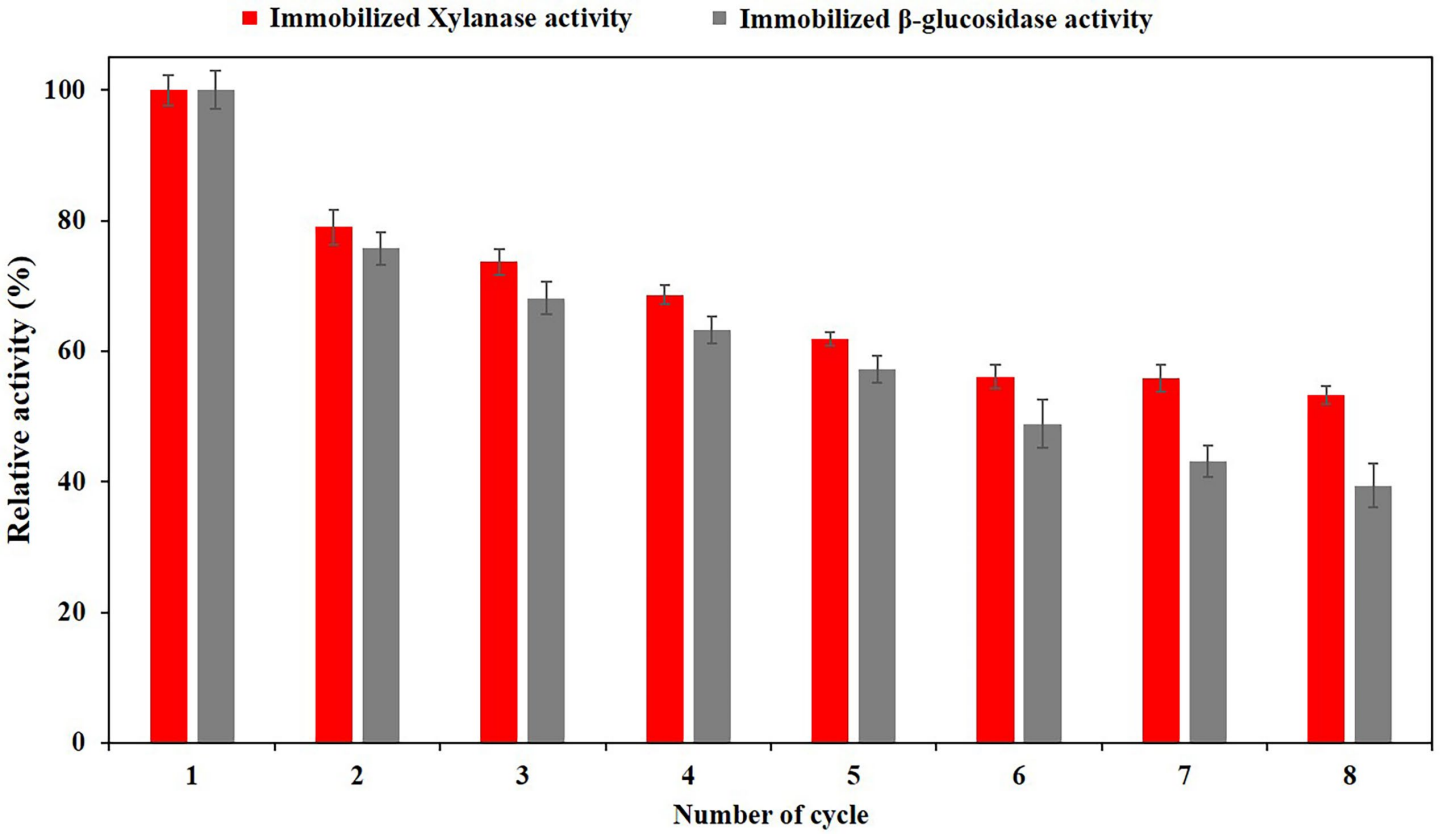
**(A)** Hydrolysis of the CRW by the free and immobilized PersiBGLXyn1 after 10 days at 50°C. **(B)** Bioethanol production from the CRW hydrolysates produced by the free and immobilized PersiBGLXyn1 during 3 days of fermentation.

Lignocellulosic biomass is the major source of fermentable sugars. On the other hand, enzymatic biodegradation of these materials has gained much interest in recent years due to their potential in producing sustainable biofuels. Furthermore, using the pretreated lignocellulosic biomass can facilitate enzymatic hydrolysis by enhancing cellulose and hemicellulose content and lignin reduction (Bala and Singh, 2019). To reveal the hydrolytic properties of the free and immobilized PersiBGLXyn1, they were used for the hydrolysis of CRW. The high polysaccharides content of CRW is mainly composed of cellulose and hemicellulose. Furthermore, it can be a suitable substrate for cellulases and hemicellulase enzymes. Some studies mentioned that CRW contains nearly 80% cellulose and hemicellulose (Corrêa et al., 2021). In this study, the CRW contained 82% holocellulose, 0.27% lignin, 13.80% extractive and 0.01% ash, making it a suitable raw material to hydrolyze by bifunctional PersiBGLXyn1. Based on the results from Figure 6A, the immobilized PersiBGLXyn1 showed higher amounts of reducing sugar compared to the free one, except for 2 days of hydrolysis. After 2 days of hydrolysis, the free enzyme showed 8.01 g/l reducing sugar, while this amount was lower in the presence of the immobilized PersiBGLXyn11 (5.22 g/l). It was further observed an increase in the amount of reducing sugar produced by the immobilized PersiBGLXyn1 when compared with the free one after 4 days. The maximum amount of reducing sugars was observed after 10 days of hydrolysis was 12.97 g/l and 19.69 g/l in free and immobilized enzyme, respectively. These results displayed the effectiveness of the immobilization technique to enhance the hydrolysis ability of the bifunctional enzyme. Currently, improving the hydrolysis yield of lignocellulosic feedstock such as CRW is an effective strategy to reduce the cost of bioethanol production. In former research, biodegradation of the 1% acid pretreated CRW using cellulase (8.2 mg/g sub), pectinase (8.4 mg/g sub), and xylanase (4.8 mg/g sub) resulted in the production of 5.68 g/l reducing sugar after 72 h hydrolysis (Kim et al., 2017). In another report, using the cellulase enzyme (18.3 mg /g sub) for hydrolysis of the 1% popping pretreated CRW showed less than 4 g/l reducing sugar after 48 h (Choi et al., 2012). Likewise, conversing the alkaline pretreated coffee skin by cellulase enzyme resulted in 0.6 g/l reducing sugar after 48 h (Febrianto, 2018). Our results demonstrated the applicability of the PersiBGLXyn1 in the biodegradation of biomass and the immobilization efficiencies for various lignocellulose-based applications.

### Ethanol production

The renewable lignocellulosic agro-residues are cost-effective resources for bioethanol production. In the current study, to assess the capability of the free and immobilized PersiBGLXyn1 for ethanol production, the CRW was used. The total ethanol production titre on the 1 day of fermentation was found to be 20.19 g/l and 31.91 g/l for the free and immobilized enzyme, respectively (Figure 6B). The bioethanol produced after 48 h was higher for the pretreated biomass treated with the immobilized enzyme and showed 37.60 g/l, while this amount was 25.21 g/l for the free PersiBGLXyn1. In the final experiment time, the ethanol production titre was 29.31 g/l and increased remarkably in the immobilized sample (51.47 g/l).

Numerous studies have been performed on the use of CRW in ethanol production titre Table 3 shows the comparison between the amount of ethanol produced by CRW in enzymatic hydrolysis and fermentation by different cellulose and hemicellulose enzymes with free and immobilized PersiXynBGL1, and as can be seen, PersiXynBGL1 had higher bioethanol production.

**Table 3:** Comparison of ethanol yields obtained after enzymatic hydrolysis and fermentation by different cellulase and hemicellulose enzymes with the free and immobilized PersiXynBGL1.

Biomass	Pretreatment	Enzyme	Fermentation condition	Ethanol (g/L)	Ref.
Coffee husk waste	dilute acid hydrolysis-alkaline	Cellulase	*S. cerevisiae-32* h at 30°C	48.19	(Morales-Martínez et al., 2020)
Coffee cherry husk	Steam	Cocktail of cellulase, xylanase, pectinase, mannanase and laccase	Immobilized *yeast S. cerevisiae-72* h	2.19	(Shankar et al., 2019)
Coffee residue waste	popping	Cellulase	*S. cerevisiae-96* h at 37°C	15.3	(Choi et al., 2012)
Spent coffee	Acid	Cellulase / Betaglucosidase	*S. cerevisiae-12* h at 37°C	47.9	(Dadi et al., 2018)
Coffee pulp	Alkali	Cellulase	*S. cerevisiae-48* h at 30°C	11.99	(Menezes et al., 2014)
Coffee residue waste	Organic solvent	In-house produced cellulase and pectinase	*S. cerevisiae-12* h at 30°C	10.4	(Nguyen et al., 2017)
Coffee residue waste	Organic solvent	Free and immobilized bifunctional PersiXynBGL1	*S. cerevisiae-72* h at 28°C	Free: 29.33 g/l Immobilized: 51.43 g/l	This study

According to Table 3, compared with former studies, the ethanol production from the alkaline pretreated coffee pulp using cellulase enzyme obtained 11.99 g/l ethanol after 48 h fermentation which was lower than the ethanol produced free PersiBGLXyn1 (Menezes et al., 2014). In another study, hydrolysis of the organic solvent pretreated CRW by cellulase and pectinase showed 10.40 g/l ethanol production after 12 h (Nguyen and Kim, 2017). In other studies that have been done to produce ethanol by enzymatic digestion and fermentation, after different pretreatment methods on CRW, the amount of ethanol produced is less than the immobilized PersiBGLXyn1 due to the time, and amount of CRW consumed (Choi et al., 2012; Shankar et al., 2019).

However, the differences between ethanol production yields are due to the various pretreatments and reaction conditions used in different studies. It can be observed that the bioethanol produced during the fermentation in the sample treated with immobilized PersiBGLXyn1 starting with an initial concentration of 31.91 g/l at 24 h and reaching 51.47 g/l at the end of fermentation, whereas this value was 29.31 g/l in sampled treated with free PersiBGLXyn1. This represents the potential of the immobilized PersiBGLXyn1 as a promising alternative for improved fermentation efficiency of yeast and enhanced converting CRW, a primary industrial waste, to renewable biofuel.

This study used a lab-scale fermentation system for bioethanol production from the CRW. The laboratory-scale results gave valuable information about the potential of the immobilized metagenome-derived PersiBGLXyn1 in lignocellulose conversion and ethanol production. For the feasibility of enzyme transfer in industrial lignocellulosic ethanol processes, further studies must be conducted at full scale. However, due to the complexity of the large-scale operations, more investigations should be carried out to meet the requirements of industrial-scale production.

## Conclusion

In recent years, some enzymes as biocatalysts have played multiple physiological roles, known as multi-functional enzymes. More multi-functional enzymes are being discovered thanks to sequencing technologies and metagenomics advances. In this study, a novel bifunctional xylanase/β-glucosidase metagenomic-derived enzyme with underground β-glucosidase activity was mined by in-silico screening, cloned, expressed and purified and finally named PersiBGLXyn1. The bifunctional PersiBGLXyn1was immobilized on NC to improve the enzyme’s underground β-glucosidase activity and higher stability. The immobilized enzyme exhibited higher reusability, activity, and stability on pH and temperatures; hence, it is possible to use it in biomass-based applications. Due to its affluence in cellulose and hemicellulose, the CRW was a potential bioethanol production substrate. Therefore, the capability of bifunctional enzymes for converting CRW into reducing sugar and subsequent bioethanol production was evaluated. The better performance of the immobilized enzyme over the free form in improving the underground activity led to the higher production of fermentable sugars and bioethanol.

## Data availability statement

The original contributions presented in the study are included in the article/Supplementary material; further inquiries can be directed to the corresponding authors.

## Author contributions

EM: synthesis of support nano-carriers, conceptualization, methodology, and writing—original draft. KK: contributed to the computational, bioinformatics data analysis, and writing—review and editing. ReG: biochemical characterization and writing—original draft. RaG: investigation and writing—original draft. SA: conceptualization, methodology, writing—original draft, and resources. GS: supervision, project administration, and writing—review and editing. BZ and SR: computational data analysis and editing—revised manuscript. All authors contributed to the article and approved the submitted version.

## Funding

This research was supported by grants from Agricultural Biotechnology Research Institute of Iran (ABRII).

## Conflict of interest

The authors declare that the research was conducted in the absence of any commercial or financial relationships that could be construed as a potential conflict of interest.

## Publisher’s note

All claims expressed in this article are solely those of the authors and do not necessarily represent those of their affiliated organizations, or those of the publisher, the editors and the reviewers. Any product that may be evaluated in this article, or claim that may be made by its manufacturer, is not guaranteed or endorsed by the publisher.
