# Supplementary figures and images for: Enhancing the ethanol production by exploiting a novel metagenomic-derived bifunctional xylanase/β-glucosidase enzyme with improved β-glucosidase activity by a nanocellulose carrier
Source: Front Microbiol. 2023 Jan 4;13:1056364. doi: 10.3389/fmicb.2022.1056364 (PMC9845577; doi:10.3389/fmicb.2022.1056364)

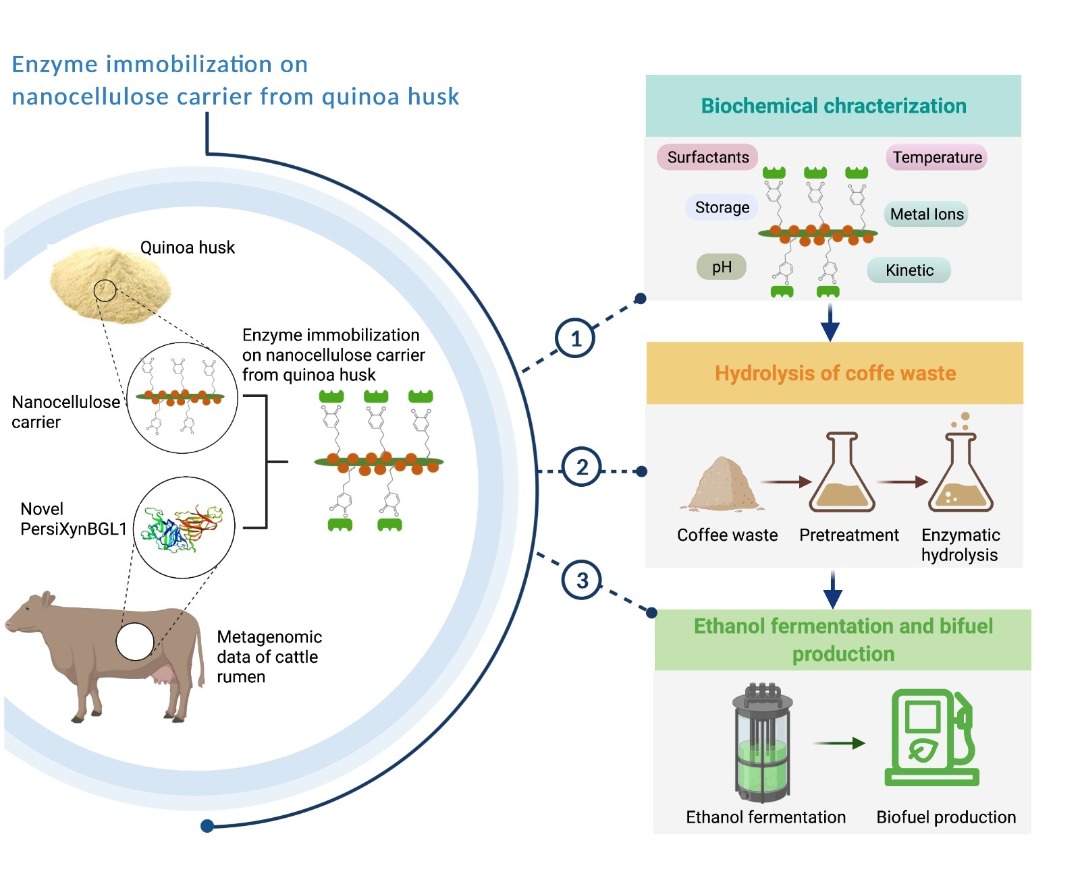

Supplement: Supplementary file 1 [file Image_1.JPEG]
